# Retraction: Magnetic nanoparticles modified with a copper(i) complex as a novel and efficient reusable catalyst for A^3^ coupling leading to C–N bond formation

**DOI:** 10.1039/d5ra90059j

**Published:** 2025-05-19

**Authors:** Wei Li, Jinlong Yan, Wenjing Xu, Li Yan Zhang

**Affiliations:** a College of Science and Engineering, Jiaozuo Normal College Jiaozuo Henan 454000 China 1295007008@jzsz.edu.cn; b Institute Chemical Technology Guangzhou China liyanzhang9090@gmail.com

## Abstract

Retraction of ‘Magnetic nanoparticles modified with a copper(i) complex as a novel and efficient reusable catalyst for A^3^ coupling leading to C–N bond formation’ by Wei Li *et al.*, *RSC Adv.*, 2023, **13**, 28964–28974. https://doi.org/10.1039/D3RA04871C.

The Royal Society of Chemistry hereby wholly retracts this *RSC Advances* article due to concerns with the reliability of the data.

There are duplications of multiple NMR spectra with another paper published by different authors.^1^ The authors were contacted but did not provide a response to the concerns.

Given the significance of these concerns and lack of raw data, the Editor has lost confidence that the findings presented in this paper are reliable.

The authors were informed about the retraction of the article, Li Yan Zhang has responded but not indicated if they agree with the decision to retract, no other authors responded.

Signed: Laura Fisher, Executive Editor, *RSC Advances*

Date: 9th May 2025
